# Editorial: Endocrine disruption in marine species: unraveling pollution and climate change effects

**DOI:** 10.3389/fendo.2025.1616931

**Published:** 2025-05-08

**Authors:** Juan Ignacio Bertucci, Ayelén Melisa Blanco, M. Dulce Estêvão

**Affiliations:** ^1^ Centro Oceanográfico de Vigo, Instituto Español de Oceanografía – CSIC, Vigo, Spain; ^2^ Centro de Investigación Mariña, Laboratorio de Fisioloxía Animal, Departamento de Bioloxía Funcional e Ciencias da Saúde, Facultade de Bioloxía, Universidade de Vigo, Vigo, Spain; ^3^ Universidade do Algarve, Escola Superior de Saúde, Faro, Portugal; ^4^ Algarve Biomedical Center Research Institute (ABC-Ri), Universidade do Algarve, Faro, Portugal

**Keywords:** endocrine disruptors, marine contamination, climate change, environmental endocrinology, molecular endocrinology, marine toxicology

## Introduction

Endocrine disruption in marine species has emerged as a significant concern in environmental endocrinology, particularly in the context of escalating anthropogenic pressures. Persistent pollutants, including microplastics, heavy metals, and agrochemical residues, alongside climate-induced stressors like ocean warming and acidification, are now recognized as potent modulators of endocrine function in aquatic organisms. These stressors compromise critical physiological and behavioral processes, with potential implications for individual fitness, population viability, and ecosystem stability.

The Research Topic *Endocrine disruption in marine species: unraveling pollution and climate change effects* brings together a multidisciplinary set of contributions that examine the mechanistic underpinnings, organismal impacts, and ecological implications of endocrine disruption across marine taxa. This editorial synthesizes the key findings, contextualizes them within the broader scientific discourse, and highlights knowledge gaps and future research directions.

## Aims and scope of the Research Topic

The central objective of this Research Topic is to advance the mechanistic understanding of how environmental contaminants and climate-related stressors might perturb endocrine pathways in marine organisms. The articles included address fundamental questions including:

- What molecular and cellular mechanisms underlie endocrine disruption in marine systems?
- How are physiological endpoints such as growth, reproduction, and stress response altered by exposure to endocrine-disrupting chemicals?
- In what ways do environmental parameters associated with climate change interact with or potentiate these disruptions?
- What strategies can be implemented to mitigate endocrine-disruptive effects and reduce pollutant input into marine environments?

This Research Topic emphasizes the importance of interdisciplinary approaches, bridging molecular endocrinology, marine toxicology, ecophysiology, and environmental policy.

## Thematic integration of contributions

Yadetie et al. conducted a study exploring the effects of two endocrine-disrupting chemicals, ethynylestradiol and bisphenol A (BPA, on hormonal and metabolic pathways in the pituitary and liver of female Atlantic cod. The researchers found that both chemicals significantly impact reproductive and metabolic functions. They observed that the expression of key genes involved in hormonal regulation and metabolic homeostasis was altered in the pituitary, while the liver exhibited changes related to triglyceride synthesis, highlighting the systemic influence of these contaminants.

Thomas et al., in a perspective article, critically examined the environmental and human health risks associated with sunscreen ingredients. They raise concerns over chemicals, such as butylparaben, oxybenzone, and octinoxate, emphasizing their potential to harm marine ecosystems and pose risks to human health. The article also discusses the ongoing debate between chemical-based and mineral-based sunscreens in terms of safety and effectiveness, advocating for more sustainable and health-conscious choices.

Brun et al. investigated the molecular responses of two sub-Arctic fish species, capelin and long rough dab, when exposed to benzo[a]pyrene, a toxic compound found in oil. Using liver slice cultures and RNA sequencing, the study revealed notable gene expression changes, especially affecting detoxification pathways such as the aryl hydrocarbon receptor pathway. The differential responses observed in these species, compared to polar cod, are linked to physiological and lipid content differences. This research provides valuable biomarkers and genomic insights for monitoring the impact of oil pollution in cold-water marine species.

Fodor et al. examined whether mollusks possess functional membrane sex steroid receptors akin to those found in vertebrates. Using the great pond snail *Lymnaea stagnalis* as a model, they identified putative receptor gene sequences but found no evidence of binding activity with vertebrate sex steroids, such as estrogen, progesterone, or testosterone. The absence of rapid endocrine signaling responses suggests that such receptor-based steroid signaling may be unique to chordates and is likely not conserved in mollusks.

Shi et al. focused on the black tiger shrimp (*Penaeus monodon*) and their molecular response to acute low-salinity stress. Employing high-throughput sequencing, the researchers identified 167 microRNAs showing significant expression changes under stress conditions. Most miRNAs were downregulated, indicating gene activation for adaptive responses. Enrichment analyses pointed to critical roles of the nervous, immune, and endocrine systems in maintaining homeostasis, providing new genetic insights for improving shrimp resilience in aquaculture.

## Broader implications and future directions

The research compiled in this Topic upholds the ecological and evolutionary significance of endocrine disruption in marine systems. Given the central role of hormones in homeostatic regulation, disruptions at this level can reverberate through biological hierarchies, from molecular signaling to community dynamics. Moreover, the combined effects of contaminants and climate stressors may act synergistically, reducing the adaptive capacity of marine organisms.

As a brief closing remark to this Research Topic, it is important to highlight that future studies should prioritize the following areas to further complement this field of investigation:

- Longitudinal and multigenerational studies to assess chronic effects and transgenerational inheritance of endocrine alterations;
- Systems-level analyses integrating omics approaches for biomarker discovery;
- Ecosystem-based modelling to predict population and community-level outcomes;
- Policy-relevant frameworks for translating scientific findings into regulatory actions.

## Conclusion

*Endocrine disruption in marine species: unraveling pollution and climate change effects* offers a timely synthesis of current knowledge on how anthropogenic pressures compromise endocrine function in marine organisms. By elucidating mechanisms, identifying sensitive taxa, and proposing mitigation strategies, the contributions collectively advance the field of marine ecotoxicology and support evidence-based conservation and policy-making. As anthropogenic stressors intensify, the integration of endocrine disruption into marine environmental assessments will be critical for the protection of marine biodiversity and ecosystem function.

